# Mechanisms of Alcohol-Induced Endoplasmic Reticulum Stress and Organ Injuries

**DOI:** 10.1155/2012/216450

**Published:** 2011-10-26

**Authors:** Cheng Ji

**Affiliations:** Southern California Research Center for ALPD and Cirrhosis, USC Research Center for Liver Disease, Department of Medicine, Keck School of Medicine, University of Southern California, Los Angeles, CA 90089, USA

## Abstract

Alcohol is readily distributed throughout the body in the blood stream and crosses biological membranes, which affect virtually all biological processes inside the cell. Excessive alcohol consumption induces numerous pathological stress responses, part of which is endoplasmic reticulum (ER) stress response. ER stress, a condition under which unfolded/misfolded protein accumulates in the ER, contributes to alcoholic disorders of major organs such as liver, pancreas, heart, and brain. Potential mechanisms that trigger the alcoholic ER stress response are directly or indirectly related to alcohol metabolism, which includes toxic acetaldehyde and homocysteine, oxidative stress, perturbations of calcium or iron homeostasis, alterations of S-adenosylmethionine to S-adenosylhomocysteine ratio, and abnormal epigenetic modifications. Interruption of the ER stress triggers is anticipated to have therapeutic benefits for alcoholic disorders.

## 1. Introduction

**Figure 1:**
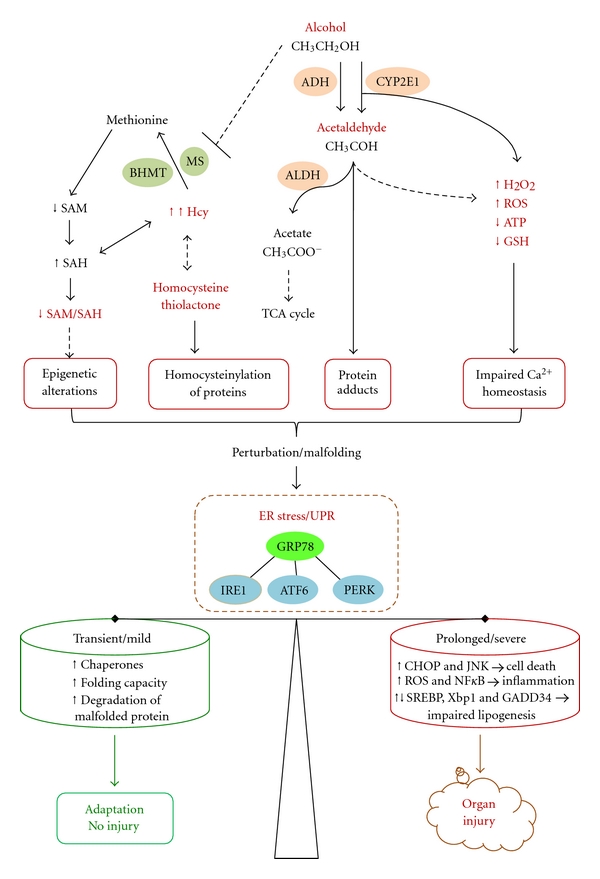
Mechanisms of alcohol-induced endoplasmic reticulum (ER) stress and organ injuries. ADH: alcohol dehydrogenase; ALDH: acetaldehyde dehydrogenase; CYP2E1: cytochrome P450 2E1; ROS: reactive oxidative stress; GSH: glutathione; BHMT: betaine-homocysteine methyltransferase; MS: methionine synthase; Hcy, homocysteine; SAM: S-adenosylmethionine, SAH: S-adenosylhomocysteine; TCA: tricarboxylic acid; UPR: unfolded protein response; GRP78: glucose-regulated protein 78; IRE1: inositol requiring enzyme; ATF6:activating transcription factor 6; PERK: protein kinase ds RNA-dependent-like ER kinase; CHOP: C/EBP-homologous protein; JNK, c-jun-N-terminal kinase; NF*κ*B, nuclear factor *κ*B; SREBP: sterol regulatory element binding protein; Xbp-1: X box binding protein 1; GADD34: growth arrest and DNA damage-inducible protein. See the context for details.

Alcohol is the most socially accepted addictive drug. Alcohol abuse and dependence causes social problems such as domestic violence and loss of productivity in work place as well as traffic accident-related injuries and chronic organ disorders. Excessive alcohol use is the third leading cause of preventable death in the United States and is responsible for 3.8% of deaths worldwide [1–3]. Alcohol-related medical problems can be improved upon a good understanding of pathogenesis of alcohol-induced injuries. After its consumption, alcohol is readily distributed throughout the body in the blood stream and crosses biological membranes which affect virtually all organs and biological processes in the body. Most of the alcohol that enters the body is first oxidized to toxic acetaldehyde, which is catalyzed by the cytosolic alcohol dehydrogenase (ADH) (Figure 1). Acetaldehyde is then converted by acetaldehyde dehydrogenase (ALDH) to acetic acid, which occurs primarily in the liver [4]. Alcohol can also be oxidized to acetaldehyde by cytochrome P450IIE1 (CYP2E1) which generates hydrogen peroxide. Alcohol-related medical illness results directly or indirectly from the toxic alcohol metabolites in cells and tissues. Alcoholic injuries can be found in most organs including brain, gastrointestinal tract, immune system, kidney, lung, heart, pancreas, and most frequently liver (reviewed in [1, 5–13]). Alcohol-induced liver disease (ALD) is better characterized than in other organs. The progression of ALD includes a spectrum of liver diseases, ranging from steatosis, steatohepatitis, fibrosis, to cirrhosis and even cancer [1, 7, 13]. However, the underlying molecular mechanisms of ALD are not completely understood. Both primary factors and cofactors are involved in the pathogenesis of ALD. Primary factors include but are not limited to increased oxidative stress mainly from mitochondrial malfunction and CYP2E1, increased endotoxin production and TNF signaling, impaired innate and adaptive immunity, hypoxia, impaired methionine metabolism, and epigenetic modifications [7, 9, 10, 13–18]. Cofactors may include malnutrition or complications with diabetes, obesity, smoking, or HCV/HIV infections [1, 9, 10, 13]. Alcohol-induced perturbations of homeostasis in the endoplasmic reticulum (ER) have evolved as an important factor contributing to fatty liver disease, which has been reviewed by a few comprehensive reviews [19–22]. Evidence for the involvement of ER in the pathogenesis of alcoholic injury is now accumulating beyond the liver. The purpose of this review is to highlight phenomenological evidence for alcohol-induced ER stress in select organ disorders and to discuss potential molecular mechanisms causing alcoholic ER stress.

## 2. ER Stress and the Unfolded Protein Response (UPR)

The ER is an essential organelle for protein synthesis and modifications, for storing and releasing Ca^2+^, for the biosynthesis of lipids and sterols, and for detoxification of certain drugs. ER stress is a condition under which unfolded or malfolded proteins accumulate in the ER (reviewed in [18–21]). ER stress results from perturbations in ER homeostasis such as calcium depletion, inhibition of glycosylation, alterations of the redox state, or lipid overloading. ER stress triggers the unfolded protein response (UPR), which constitutes a series of ER-to-nucleus signaling mediated by three ER resident transmembrane sensor proteins, inositol requiring protein 1 (IRE1), ds-RNA-activated protein kinase (PKR) like ER kinase (PERK), and activating transcription factor 6 (ATF6) (Figure 1). The three sensors are activated upon dissociation from their inhibitory binding with the chaperone GRP78/BiP. IRE1, which has kinase and endoribonuclease activities, is activated by transautophosphorylation. The activated IRE1 processes the transcription factor X-box binding protein-1 (XBP1) mRNA via the unconventional splicing to form transcriptionally active spliced XBP1 (sXBP1). sXBP1 activates UPR target genes, including chaperones and ER-associated degradation (ERAD) pathway genes. The second sensor PERK phosphorylates the eukaryotic initiation factor 2*α*-subunit (eIF2*α*), leading to an inhibition of the initiation of translation and a global attenuation in protein translation. Phosphorylation of eIF2*α* selectively activates activating transcription factor 4 (ATF4), which regulates ER chaperone genes, ERAD pathway genes, amino acid metabolism genes, and the transcription factor C/EBP homologous protein (CHOP) [19–21]. The third sensor ATF6 is cleaved in the Golgi to form a transcriptionally active fragment that traffics to the nucleus to activate UPR target genes. In general the UPR results in reduced synthesis of nascent proteins, increased unloading of unfolded proteins, and increased capacity of folding, which lead to restoration of ER homeostasis. 

However, prolonged or severe UPR provokes a complex network of interacting and parallel responses contributing to pathological consequences such as apoptosis, inflammation, and fat accumulation [19–24]. The ER stress-induced apoptosis is mediated by a few factors. CHOP regulates growth arrest and DNA damage-inducible protein (GADD34). GADD34 binds protein phosphatase-1 and enhances eIF2*α* dephosphorylation, leading to premature restoration of translation and enhanced ER stress. CHOP can also regulate expression of the TRAIL receptor DR5, pro- and antiapoptotic Bcl-2 family protein Bim, Bax and Bcl-2 modulating cell death [19–21]. Sustained activation of IRE1 recruits the adaptor protein TRAF2 and activates JNK and NF-*κ*B, both of which mediate apoptosis [23]. In addition, alterations in ER calcium homeostasis, upregulation of ER oxidase 1 (ERO1) by CHOP, activation of caspase 12, and activation of GSK3*β* by tribbles 3 (TRB3) and AKT are other mechanisms underlying ER stress-induced inflammation and apoptosis [21, 23, 25]. Lipid accumulation is also a main pathological feature of prolonged ER stress, and each of the three ER sensor pathways has direct molecular effects on lipid synthesis. The IRE1*α*-XBP1 branch regulates C/EBP*α* and C/EBP*β* that control directly the expression of genes involved in *de novo *fatty acid biosynthesis [26]. The ATF6 branch is involved in phospholipid biosynthesis as well as in fatty acid oxidation and lipoprotein secretion [27, 28]. The PERK-eIF2*α* branch influences expression of C/EBP family and PPAR*γ* transcription factors via the eIF2*α*-specific phosphatase GADD34 and regulates SREBP1-related *de novo *lipid synthesis and accumulation [18–24, 29, 30].

## 3. ER Stress in Alcoholic Organ Injuries

### 3.1. Liver

Alcohol is mainly metabolized in the liver, and liver cells are rich in ER which assumes synthesis of a large amount of secretory and membrane proteins [19, 20, 29]. Partial role of ER in alcohol metabolism was initially realized decades ago as NADH from the hepatic oxidation of ethanol to acetaldehyde by ADH was found to support also microsomal ethanol oxidations [14, 15]. The inducible microsomal ethanol oxidizing system (MEOS) is associated with proliferation of the ER and a concomitant induction of cytochrome P4502E1 (CYP2E1) in rats and in humans. Free radical release as a consequence of CYP2E1 function in the ER and subsequent oxidative stress and lipid peroxidation generally contribute to ALD [14, 15]. However, alcohol-induced ER stress response was not recognized until recently. Molecular evidence for an impaired UPR was first found in the intragastric alcohol-fed mice using microarray gene expression profiling [18]. The alterations of selected ER stress markers were associated with severe steatosis, scattered apoptosis, and necroinflammatory foci. Moderate upregulation of expression of SREBP-1c and SREBP-2 and their responsive genes was detected by immuoblotting [18]. SREBP-1c knockout mice were protected against triglyceride accumulation [30–32]. Knocking out CHOP resulted in minimal alcohol-induced apoptosis in mouse liver [32–34]. In a setting of alcohol infusion and moderate obesity, there are synergistic effects of accentuated ER and mitochondrial stress, nitrosative stress mediated by M1 macrophage activation, and adiponectin resistance on hepatic necroinflammation and steatohepatitis [35]. In micropigs fed alcohol, liver steatosis and apoptosis were shown to be accompanied by increased mRNA levels of CYP2E1, GRP78 and SREBP-1c, and protein levels of CYP2E1, GRP78, activated SREBP and caspase 12 [36]. In addition, the ER stress response was correlated with elevated transcripts of lipogenic enzymes such as fatty acid synthase (FAS), acetyl-CoA carboxylase (ACC), and stearoyl-CoA desaturase (SCD). Further, alcohol-induced lipopolysaccharide (LPS) is linked to impaired UPR and advanced hepatic injury [37–39]. In cirrhotic rat livers, only eIF2*α* was activated in the basal state. After LPS challenge, full UPR as indicated by activation of IRE1*α*, ATF-6, and eIF2*α* was detected [37]. However, LPS-induced accumulation of NF-*κ*B-dependent antiapoptotic proteins was not observed, suggesting that the UPR sensitized the cirrhotic livers to LPS/TNF*α*-mediated apoptosis. Alcohol-induced hepatic ER stress response not only occurs in rodents but also in livers of baboon and human patients [40, 41]. In baboon fed alcohol orally, upregulation of calpain 2, calpain p94, and ERD21 and downregulation of eIF2*α* were among the genes of altered expression that was revealed by using cDNA array analysis [41]. Gene expression profiling of cirrhotic liver samples from human alcoholics also revealed alterations of calpain and calreticulin that are indicative of ER malfunction.

### 3.2. Pancreas

The pancreas is one of the important digestive organs adversely affected by alcohol abuse. Pancreatitis is among the most common alcohol-related hospital diagnosis in USA [11]. The underlying mechanisms for alcohol-induced pancreatitis are not well understood. Similar to the liver, the pancreas has the capacity to metabolize alcohol via both the oxidative and nonoxidative pathways yielding toxic metabolites such as acetaldehyde and lipid esters. Fatty acid ethyl and cholesteryl esters are known to accumulate in the acinar cell after chronic alcohol consumption which decreases the stability of the membranes of zymogen granules and lysosomes [42, 43], which cause a premature activation of intracellular digestive enzyme and may predispose the gland to autodigestive inflammation and injury. In respect to the role of organelles in alcoholic pancreatic injury, the ER has been considered as the acinar cell has the highest rate of protein synthesis among all tissues in adult organism. In fact, perturbations of ER homeostasis are found in acute pancreatitis [44, 45], and all the three ER stress/UPR transducers (i.e., IRE1, ATF6, and PERK) and their downstream pathways are activated. However, chronic alcohol feeding alone causes minimal pancreatic tissue injury in animal models [45, 46]. Further studies demonstrate that alcohol feeding activates the UPR in pancreas with upregulation of the transcription factor XBP1 in the intragastric alcohol infusion model [47, 48]. This suggests that alcohol induces a physiologic adaptive UPR that may prevent pathophysiologic pancreatitis responses. Indeed, heterozygous deletion of the XBP1 gene prevents XBP1 upregulation and results in pathologic changes including extensive dilation of the ER with occasional dense luminal inclusions, hallmarks of ER stress, and significant accumulation of autophagic vacuoles in acinar cells [48]. Thus, impaired UPR in the pancreas can potentiate alcohol-induced toxicity and aggravate pancreatic damages.

### 3.3. Brain

Alcohol exposure during development has devastating effects on the loss of neurons in selected brain areas, which leads to profound damages to the central nervous system (CNS). Alcohol consumption during pregnancy causes fetal alcohol spectrum disorders (FASDs) [1, 49]. Microcephaly, abnormal cortical thickness, reduced cerebral white matter volume, ventriculomegaly, and cerebellar hypoplasia are the prominent CNS abnormalities in FASDs. Children with (FASD) have a variety of cognitive, behavioral, and neurological impairments [49]. What cause ethanol-induced neurodegeneration are not clear. Considering that ER stress plays a role in the pathogenesis of several popular neurological diseases such as Huntington's disease, brain ischemia, Alzheimer's disease, and Parkinson's disease [50–53], an involvement of ER stress in alcohol-induced neuron toxicity has been hypothesized [54]. Recent evidence from both *in vitro* and *in vivo* tests appears to support the assumption. Exposure of SH-SY5Y neuroblastoma cells or primary cerebellar granule neurons to ethanol alone had little effect on the expression of ER stress markers [54]; however, ethanol markedly increased the expression of GRP78, CHOP, ATF4, ATF6, and phosphorylated PERK and eIF2*α* in the presence of tunicamycin or thapsigargin, which was accompanied with increased cell death. Acute exposure of seven-day-old mice to ethanol by subcutaneous injection at a dose of 5 g/kg significantly increased ER stress response. Increase of ATF6, CHOP, GRP78, and mesencephalic astrocyte-derived neurotrophic factor as well as the phosphorylation of IRE1, eIF2*α*, PERK, and PKR were detected within 24 hours after the ethanol exposure. Further, the ethanol-induced increase in phosphorylated eIF2*α*, caspase-12 and CHOP was distributed in neurons of specific areas of the cerebral cortex, hippocampus, and thalamus. Since the age of the animals used in this experiment is equivalent to the third trimester of pregnancy in humans, the above evidence suggests that ethanol directly induce ER stress in the developing brain.

### 3.4. Heart

It is well documented that chronic heavy alcohol drinking is a risk factor for cardiovascular disorders including cardiac hypertrophy, myofibrillar disruption, reduced contractility, and decreased ejection fraction [55]. Alcohol may change the circulatory hemodynamics resulting in stress on the heart. The stressed heart demands more cardiac output which leads to compensative hypertrophic responses such as neurohormonal activation and increased growth factors and cytokines, resulting in enlarged cardiomyocytes and increased sarcomere assembly. ER stress may play a critical role in regulating protein synthesis in cardiac myocytes, and thereby produce cell enlargement and cardiac hypertrophy. Chronic alcohol consumption by FVB (Friend virus-B type) albino mice at 4% of diet for 12 weeks resulted in increased heart weight and heart-to-body weight ratio [56]. In the myocardium of the FVB mice chronically fed alcohol, GRP78, CHOP, and IRE1a protein expression levels were increased, indicative of the UPR. Class I alcohol dehydrogenase efficiently oxidizes alcohol resulting in increased production of acetaldehyde. Overexpressing alcohol dehydrogenase in the FVB mice during chronic ethanol treatment resulted in a greater UPR upregulation [56]. The finding indicates that acetaldehyde from alcohol metabolism may induce ER stress. Furthermore, overexpressing of the antioxidant protein metallothionein in FVB mice significantly reduced peak shortening and maximal shortening velocity of cardiac myocytes by LPS, which is often elevated in alcoholics [13–15, 39, 40]. In parallel, the transgenic FVB mice displayed decreased protein levels of GRP78, CHOP, PERK, and IRE1 whereas the wild type FVB displayed a significant increase in the protein levels of PERK, phospho-JNK, and phospho-p38 in the myocardium in response to LPS [56, 57].

## 4. Mechanisms of Alcohol-Induced ER Stress

### 4.1. Acetaldehyde Adducts and ER Stress

Alcohol-derived acetaldehyde is highly reactive [58–62]. At physiological temperature and pH, acetaldehyde reacts with nucleophilic groups in proteins, such as *α*-amino groups of internal lysine residues and the *ε*-amino group on the N-terminal amino acid of unblocked proteins forming unstable Schiff base acetaldehyde adducts. In addition, ethanol abuse may also lead to the formation of other types of protein adducts, such as malondialdehyde-acetaldehyde hybrids and *α*-hydroxyethyl protein-adducts. The acetaldehyde adducts initiate immunogenic reactions, cause conformational changes and inactivation of the adducted targets, or trigger aberrant protein degradation, which contribute to the development of alcoholic organ diseases (Figure 1). Malondialdehyde–acetaldehyde adduct is found to be the dominant epitope after malondialdehyde modification of proteins in atherosclerosis [63]. Antibodies to the aldehyde adducts have been detected in the serum of patients with atherosclerotic lesions and correlate with the progression of atherosclerosis. It is known that atherosclerosis develops as a result of protein unfolding and modification of protein and/or macromolecular complex function at the cellular level [63]. In supporting this, evidence for ER stress response was found in transgenic mice with cardiac overexpression of ADH that increased acetaldehyde exposure [56, 57]. The ADH transgene increased induction of IRE1, eIF-2*α*, GRP78, and CHOP and exacerbated chronic alcohol ingestion-induced myocardial dysfunction and hypertrophy. Further, in a mouse model of acute ethanol intoxication, inhibition of ADH causes downregulation of GRP78 mRNA levels [64]. This suggests a causal relationship between ethanol metabolism and ER stress response. Acetaldehyde adducts also affect ER Ca^2+^ handling in rat ventricular myocytes [65, 66], which may disturb ER calcium homeostasis playing a critical role in stress-mediated cellular injury [67]. In response to alcohol dosing *in vivo,* the actin in Type I and Type II fibre predominant muscles of rats was found to form stable covalent adducts with acetaldehyde [68]. Histochemical analysis showed that unreduced-acetaldehyde-protein adducts were located within the sarcolemmal (i.e., muscle membrane) and subsarcolemmal regions, which perturbed the membranes and increased protein and enzyme activity of sarcoplasmic-ER Ca^2+^-ATPase, resulting in muscle cell death and alcoholic myopathy. In addition, acetaldehyde adducts are found in the central nervous system which may be responsible for alcoholic ER stress response. In the brain of a heavy drinker who had died suddenly while drinking continuously, acetaldehyde adducts were immunologically identified [69]. In a mouse model administered with the Lieber-DeCarli liquid diet and alcohol, acetaldehyde adducts were readily detected in degenerated neurons in the cerebral cortex [70]. The neural region that alcoholic ER stress response occurred colocalized with the acetaldehyde adducts. In young mice, ethanol-induced increase in ER stress protein markers was found to be distributed in the immature neurons of specific areas of the cerebral cortex, hippocampus and thalamus [54]. Thus, while most organs of the body can be affected by alcohol-derived acetaldehyde, cardiac and skeletal muscle cells and neurons appear to be particularly susceptible to acetaldehyde adducts that cause ER stress and injury.

### 4.2. Homocysteine Toxicity and ER Stress

Homocysteine (Hcy) is a normal intermediate involved in the metabolism of the essential amino acid-methionine (Figure 1). Excessive Hcy is toxic to cells. An abnormally elevated level of Hcy in the blood, a medical condition termed hyperhomocysteinemia (HHcy), is an independent risk factor in cardiovascular, neurodegenerative diseases, diabetes, obesity, and hepatic steatosis [32, 71–73]. It is generally accepted that aminoacyl thioester homocysteine thiolactone (HTL) derived from Hcy editing during protein synthesis contributes to the most of Hcy toxicity [74, 75]. HTL undergoes not only nucleophilic, which can be facilitated in the presence of acetaldehyde, but also electrophilic reactions to form protein adducts or homocysteinylation of protein lysine side chains and/or other free amine groups [75]. These reactions cause malfolding of proteins and trigger ER stress response. Evidence linking HHcy to ER stress and alcoholic liver injury has well been established in cell and animal models [16, 18–20, 32]. The intragastric alcohol feeding exhibited a greater than 5-fold increase in mouse plasma Hcy [18, 34, 35]. Hcy is metabolized normally by remethylation to methionine which is catalyzed by methionine synthase (MS) using folate as a methyl donor and by betaine-homocysteine methyltransferase (BHMT) using betaine as a methyl donor. Chronic alcohol-induced disturbance of methionine metabolism appears to contribute to the alcoholic HHcy. Alcohol inhibits enzyme activity of MS in mice and rats and reduces mRNA expression of BHMT and MS in mice [16, 17, 34, 76–79]. Simultaneous betaine feeding in the intragastric alcohol-fed mice decreased alcoholic HHcy and abrogated ER stress response in parallel with decreased ALT and amelioration of alcohol-induced necroinflammation, apoptosis, and fatty liver [18]. In cultured HepG2 cells, BHMT overexpression inhibited Hcy-induced ER stress response, lipid accumulation, and cell death [77]. In primary mouse hepatocytes, suppression of BHMT by RNA interference potentiated Hcy-induced but not tunicamycin-induced ER stress response and cell injury [77]. Transgenic mice expressing human BHMT in organs peripheral to the liver are resistant to alcohol or a high methionine and low folate diet induced HHcy and fatty liver [78]. In intragastric alcohol-fed rats, BHMT is induced, which minimizes the effect of inhibited MS on Hcy levels and subsequent ER stress response and injury [79]. In a survey using 14 mouse strains, Ivan Rusyn has found that the alcoholic HHcy is correlated with alcohol-induced liver jury (personal communication, 2011). Therefore, the above several lines of evidence support Hcy toxicity as a pathogenic factor contributing to alcohol-induced disorders.

### 4.3. SAM/SAH Ratio, Epigenetic Alterations and ER Stress

There are two types of important epigenetic regulations of gene expression: DNA methylation of cytosines within CpG dinucleotides and histone modifications [80, 81]. Aberrant epigenetic changes are involved in the etiology of a growing number of disorders such as alcohol dependence. Both global hypomethylation of DNA in liver and hypermethylation of DNA from peripheral blood cells have been reported in animal models and in human subjects with alcohol dependence [82–86]. This is because DNA methylation in general depends on the methyl donor S-adenosylmethionine (SAM) and is inhibited by S-adenosylhomocysteine (SAH). Both SAM and SAH are involved in methionine metabolism [87, 88]. Inside the cell, SAM is demethylated to SAH, which is readily converted to Hcy which is remethylated to methionine. Plasma Hcy is not metabolized and represents the cumulative export of Hcy from liver and other tissues. Alcohol consumption decreases levels of SAM and increases levels of SAH and/or Hcy resulting in a decrease in SAM to SAH ratio (Figure 1) [76, 78, 87–92]. Thus, alcohol has a marked impact on the hepatic methylation capacity. Evidence demonstrating epigenetic effects on alcoholic ER stress is emerging [17, 82]. In 66 male alcoholic patients with alcohol dependence, chronically elevated Hcy levels are associated with increased DNA methylation in the promoter region of homocysteine-inducible ER protein (HERP) and decreased expression of HERP mRNA in the blood [93, 94]. The decrease in HERP levels is followed by a lethal ER stress, mitochondrial dysfunction, and cell death in neurons of the developing and adult brain [94]. Thus it is conceivable that alcoholic Hcy regulates HERP and causes ER stress and injury through an epigenetic mechanism. In respect to the epigenetic modifications of histone, it is reported that alcohol causes a dose- and time-dependent selective acetylation of histone H3-K9 in cultured hepatocytes [95, 96]. Intragastric administration of ethanol increases the levels of acetylated H3-K9 by 2-3 folds in the liver of mice after 12 h [97]. Further analysis indicates that the increased acetylation is tissue specific as it is noted in liver, lung, and spleen but not in tissues from other organs tested. Thus, while other stress pathways such as the MAPK signaling may be involved, the alcoholic epigenetic effects on the ER stress pathways can be more relevant. For instance, in both cystathionine beta synthase heterozygous (CBS^+/−^) and wild type (WT) mice fed ethanol diets by intragastric infusion for 4 weeks, steatohepatitis, reduction in liver SAM, elevation in liver SAH, and reduction in the SAM/SAH ratio were observed [17]. Hepatic ER stress markers including GRP78, ATF4, CHOP, caspase 12, and SREBP-1c were upregulated and negative correlated with the SAM/SAH ratio in response to alcohol. Further, trimethylated histone H3 lysine-9 (3meH3K9) protein levels in centrilobular regions revealed by immunohistochemistry were reduced in ethanol-fed mice. The levels of 3meH3K9 in the promoter regions of GRP78, SREBP-1c, and CHOP revealed specifically by a chromatin immunoprecipitation assay were decreased only in CBS^+/−^ mice fed alcohol. Since CBS is involved in transsulfuration of Hcy, the findings imply that interactions of CBS ablation and alcohol feeding impair methionine metabolism, which leads to epigenetic modifications of ER stress signaling pathways. In addition, the key modulator of UPR, sXBP1 has recently been found to be a nonhistone protein target of acetylation mediated by p300 and deacetylation mediated by the NAD^+^-dependent class III deacetylase SIRT1 (sirtuin 1) [98, 99]. SIRT1 is demonstrated to be one of the major targets of alcohol action which influences TNF-*α* production in macrophages and alters glucose and lipid metabolism in the liver leading to hepatic steatosis and inflammation [100–102]. SIRT1 may also play a role in alcohol-induced ER stress response and injury through an epigenetic mechanism.

### 4.4. Oxidative Stress and Disruption of Ca^2+^ or Iron Homeostasis and ER Stress

In the ER, proteins undergo oxidative protein folding. PDI is a critical oxoreductase that catalyzes disulfide bond formation with consequent generation of reactive oxygen species (ROS) during the oxidative protein folding [19, 103]. ROS is normally under control due to cellular glutathione that sustains PDI ability to regenerate and form disulfide bridges repeatedly [103–105]. However, chronic ethanol consumption increases the production of a variety of ROS, including superoxide, H_2_O_2_, lipid peroxides, and peroxynitrite [1, 13–15]. Alcoholic ROS reduce glutathione level and increase oxidized glutathione, which breaks the redox status of the ER (Figure 1). This loss of redox homeostasis perturbs the oxidative folding and makes PDI ineffective in the catalytic redox cycles leading to more utilization of reduced glutathione. Depletion of glutathione generates excessive ROS which triggers ER stress. Antioxidant treatment, CHOP deletion, or translation attenuation has been shown to reduce oxidative stress and preserve ER function [19–23]. Ethanol rapidly caused oxidative stress in cultured neuronal cells and antioxidants blocked alcoholic potentiation of ER stress and cell death [54]. An association of ER stress response with increased oxidized glutathione was found in the pancreatic acinar cell of the ethanol-fed rats [47]. In HepG2 cells, acetaldehyde impaired mitochondrial glutathione transport and stimulated mitochondrial cholesterol content, the latter of which was preceded by increased levels of CHOP and SREBP1 [106]. Chronic exposure of animals to alcohol or overexpression of cytochrome CYP2E1 in hepatocytes increases the expression of superoxide dismutase (SOD) and activates nuclear factor erythroid 2-related factor 2 (Nrf2), which is an ER stress responsive factor [14, 107–109]. These lines of evidence suggest an intimate relationship between ER stress and ROS production. Furthermore, alcoholic oxidative stress plays a critical role in possible interplays between ER stress and mitochondrial stress, which can be mediated either by intracellular calcium or iron. Alcohol or Hcy induces alterations of lipid composition in the ER and affected ratio of phosphatidylcholine (PC) to phosphatidylethanolamine (PE) [20, 78]. Alterations of the PC/PE ratio disrupt ER calcium homeostasis causing ER stress [110]. Under ER stress, abnormal release of intracellular Ca^2+^ from the ER via inositol 1,4,5-triphosphate receptor (IP3R) channels leads to excessive mitochondrial Ca^2+^ uptake, which in turn promotes ROS production and apoptosis via multiple effects on the mitochondria [67, 111, 112]. Elevated serum iron indices (transferrin saturation, ferritin) and hepatic iron overloading are often observed in patients with alcoholic liver disease [113–117]. Excessive iron damages mitochondrial iron–sulfur clusters that generate defects in heme-containing cytochrome c and cytochrome oxidase leading to excess mitochondrial ROS [118]. Iron homeostasis is regulated by hepcidin, a circulatory antimicrobial peptide synthesized in hepatocytes [119]. Critically, ER stress response can regulate expression of hepcidin [19, 29, 120]. Thus a vicious cycle exists: alcoholic ROS and/or ER stress damage mitochondria through iron, which in return augments ROS and stresses the ER further, all of which probably act synergistically to cause severe alcoholic injury.

### 4.5. Synergistic ER Stress by Alcohol, Drugs, Viral Infection and Environments

Acute alcohol or chronic alcohol at moderate concentrations may not induce readily detectable ER stress response in some cell and animal models [29, 47]. This does not rule out the doomed potential of alcohol to induce ER stress. Indeed, ER stress can be synergistically induced by alcohol in the presence of environmental factors, genetic predispositions, drugs, or virus infection. First, it is recently noted that an accelerated development of pancreatitis in alcoholic patients who smoke may result from an additive or multiplicative effect that is mediated by ER stress response [47]. Second, in a mouse model with liver-specific deletion of Grp78, low-level oral alcohol feeding did not induce HHcy that is often seen in mice fed high doses of alcohol [29]. However, the low alcohol feeding activated SREBP1 and unconventional splicing of Xbp1 (sXbp1) and decreased Insig 1 and ATF6 and its downstream targets such as ERp57 and Derl3 in the liver GRP78 knockouts, leading to aggravated lipid accumulation in the liver. Thus, compared to the aforementioned Hcy-ER stress mechanism, Grp78 deletion represents a genetic predisposition that unmasks a distinct mechanism by which alcohol induces ER stress, one that normally is largely obscured by compensatory changes in normal animals or presumably in the majority of human population who have low-to-moderate drinking. Similarly, certain drugs potentiate alcoholic ER stress response. For instance, some HIV protease inhibitors (HIV PIs) used in anti-HIV therapeutics can cause adverse side effects such as dyslipidemia and liver injury [29, 121, 122]. Portion of HIV-infected patients often concomitantly consume or abuse alcohol leading to more severe liver injury. One of the underlying mechanisms is severe ER stress responses that are caused by both alcohol and the HIV drugs. It has been demonstrated that single gavage dosing for alcohol alone or ritonavir and lopinavir combined did not induce detectable liver injury in wild type [29]. However, the gavage treatment with alcohol plus the two HIV drugs caused significant increase in plasma ALT as well as activation of CHOP, ATF4, and sXbp1. Thus, alcohol exacerbates some HIV drug-induced ER stress and subsequent injury. Third, it is known that both alcoholic activation of the ER stress sensor-IRE1*α* and alcohol-induced accumulation of proinflammatory cytokines such as TNF*α*, IL-6, and MCP-1 activate JNK and/or NF-*κ*B pathways that mediate tissue/organ injuries [9, 10, 23, 29, 37–39]. This pathway overlap may be a result of interactions between ER stress and inflammation. The likely scenario is that mild ER stress under moderate alcohol dosing has a negative impact on ER function, which makes cells more susceptible to inflammatory signals, which subsequently augments ER stress response and injury via the JNK pathway. Fourth, alcohol may sensitize virus-infected cells to ER stress and apoptosis. It is reported that hepatitis C (HCV) infection causes ER stress in cell and animal models as well as in patients with chronic HCV [123–125]. HCV directly induces steatosis and development of hepatocellular carcinoma (HCC), which is correlated with a state of oxidative stress in mice transgenic for the HCV core protein [126, 127]. There is clinical evidence indicating that alcohol metabolism increases HCV replication and modulates the host response to HCV [128, 129]. The HCV nonstructural protein 5A (NS5A) localizes to the ER and is part of the HCV replication complex that forms altered cytoplasmic membrane structures. The membrane structure triggers ER stress and the UPR, leading to a release of ER Ca^2+^ stores and subsequent oxidative stress [124]. In addition, interactions between HCV core and destabilization of the mitochondrial electron transport chain result in increased production of ROS [130, 131]. Since alcohol alone perturbs Ca^2+^ homeostasis and stimulates ROS generation, it is conceivable that ROS mediates the synergistic interactions between alcohol consumption and HCV infection.

## 5. Concluding Remarks

While a large number of different stress responses and pathological pathways have been implicated in ethanol-induced injury [1, 7, 13–15], the occurrence of ER stress in the major organs including liver, brain, pancreas, and heart firmly supports its contributing role to alcoholic disorders. Alcohol causes alterations in many specific steps involved in the ER stress and UPR. The potential causes for alcohol-induced ER stress are directly or indirectly related to alcohol metabolism, which include but may not be limited to toxic acetaldehyde and homocysteine modifying proteins, oxidative stress from impaired CYP2E1 function and perturbations of calcium or iron homeostasis, alterations of SAM to SAH ratio and subsequent biochemical or epigenetic modifications, and, most importantly, interactions between these factors. Each of the factors may contribute more or less to the induction of the ER stress depending on tissues/organs or experimental models, dosage and duration of alcohol exposure, and presence of other environmental factors. Current investigations and conclusions on alcoholic ER stress appear depending on positive identifications of selective molecular markers of ER stress response, conclusions from which can be misleading sometimes. For instance, the ER stress-induced UPR is dynamic. It can be protective when most of the ER markers are positively detected or detrimental when most markers are latent or disappearing. The timing and quantity of the protection cannot be defined currently. Thus, circumstantially negative observations of the ER stress markers may not necessary rule out an existence of alcoholic ER stress. Future research should be directed at developing sensitive markers, particularly epigenetic markers, for identifying the alcoholic ER stress, and at defining timing and dynamics of the alcoholic ER stress and injuries using both acute and chronic models. Another point is that the ER is a cytosolic network that communicates readily with other cellular loci such as mitochondria, lysosome, cytoplasm, and nucleus. Simultaneous appearance of alcoholic dysfunctions of the other loci such as ATP depletion, abnormal degradation of the inside materials, oxidative stress, and numerous other stress responses could overshadow the role of ER stress in alcoholic diseases. Thus, the role of alcoholic ER stress in organ disorders can be defined precisely by studying complex interplays among the organelles and loci in disease pathogenesis, which could provide better therapeutic strategies targeting the ER. Finally, with respect to the therapeutic interventions at alcoholic ER stress, possible approaches include lowering homocysteine and raising SAM by nutritional support with betaine or folate [16, 20, 32], improving protein folding by using chemical chaperone PBA (sodium 4-phenylbutyrate) and TUDCA [19, 20, 29], blocking eIF2*α* dephosphorylation by using salubrinal [132], and ameliorating ROS production from the oxidative protein folding by using antioxidants. However, results of clinical trials are not available. Each of the individual approaches alone may not be a simple or universal cure as alcohol-induced pathogenesis is very complex. It is anticipated that properly combined treatments with all the beneficial agents can be effective.
